# Enhancing navigation in biomedical databases by community voting and database-driven text classification

**DOI:** 10.1186/1471-2105-10-317

**Published:** 2009-10-03

**Authors:** Timo Duchrow, Timur Shtatland, Daniel Guettler, Misha Pivovarov, Stefan Kramer, Ralph Weissleder

**Affiliations:** 1 Center for Molecular Imaging Research, Massachusetts General Hospital, Harvard Medical School, Charlestown, MA, USA; 2 Center for Systems Biology, Massachusetts General Hospital, Department of Systems Biology, Harvard Medical School, Boston, MA, USA; 3 Technische Universität München, Institut für Informatik/I12, Garching, Germany; 4 Current address: Beckman Coulter Genomics Inc., 500 Cummings Center, Suite 2450, Beverly, MA, USA; 5 Current address: German Research Center for Artificial Intelligence (DFKI GmbH), Robert-Hooke-Str. 5, 28359 Bremen, Germany

## Abstract

**Background:**

The breadth of biological databases and their information content continues to increase exponentially. Unfortunately, our ability to query such sources is still often suboptimal. Here, we introduce and apply community voting, database-driven text classification, and visual aids as a means to incorporate distributed expert knowledge, to automatically classify database entries and to efficiently retrieve them.

**Results:**

Using a previously developed peptide database as an example, we compared several machine learning algorithms in their ability to classify abstracts of published literature results into categories relevant to peptide research, such as related or not related to cancer, angiogenesis, molecular imaging, etc. Ensembles of bagged decision trees met the requirements of our application best. No other algorithm consistently performed better in comparative testing. Moreover, we show that the algorithm produces meaningful class probability estimates, which can be used to visualize the confidence of automatic classification during the retrieval process. To allow viewing long lists of search results enriched by automatic classifications, we added a dynamic heat map to the web interface. We take advantage of community knowledge by enabling users to cast votes in Web 2.0 style in order to correct automated classification errors, which triggers reclassification of all entries. We used a novel framework in which the database "drives" the entire vote aggregation and reclassification process to increase speed while conserving computational resources and keeping the method scalable. In our experiments, we simulate community voting by adding various levels of noise to nearly perfectly labelled instances, and show that, under such conditions, classification can be improved significantly.

**Conclusion:**

Using PepBank as a model database, we show how to build a classification-aided retrieval system that gathers training data from the community, is completely controlled by the database, scales well with concurrent change events, and can be adapted to add text classification capability to other biomedical databases.

The system can be accessed at .

## Background

We have previously developed a peptide database (PepBank [1]) as a repository to identify peptide based targeting ligands, peptidomimetic drugs, biological interactors, and imaging agents. Some contents of PepBank stem from public data sources; however, the major part was extracted by text mining from MEDLINE abstracts. Most of the entries are not manually curated, hence, potentially useful data are not automatically extracted from associated texts. Consequently, it is often hard to find relevant peptides without manually examining a large number of associated abstracts. This exemplifies a constraint of many modern biological databases and represents a bottleneck in more sophisticated analysis.

Biological end-users are often interested in identifying "hits" that relate to a specific disease (e.g., cancer), a disease process (e.g., angiogenesis) or a specific application category (e.g., molecular imaging). Using PepBank as a model, we defined categories that are highly relevant to a large number of biological end-users of peptide information (Table 1). We selected frequently used, broad categories based on (a) the analysis of the user queries recorded in the database log files, and the corresponding article abstracts, and (b) feedback from end users. Our users predominantly retrieved abstracts falling into categories such as those related to cancer [2], cardiovascular disease [3], diabetes [4], angiogenesis [5], apoptosis [6], molecular imaging related [7], and abstracts that have binding data available [8]. While there were many other frequently used categories, we selected a total of seven for the current study. We continue to monitor the types of queries users submit to PepBank and will add new categories as required. This includes adding more specific categories, e.g., related to subfields within oncology research. For each category, any given PepBank entry can be classified as belonging to either of two classes: *related *or *unrelated *to the category.

**Table 1:** Categories selected for the retrieval heat map.

**Category description**	**Short name**
Cancer related	CA
Cardiovascular disease related	CVD
Diabetes related	DM
Apoptosis related	APO
Angiogenesis related	ANG
Molecular imaging related	MI
Binding data available (numeric quantification of affinity)	BD

Our aim was to provide a more natural and interactive way of searching, browsing, and contextualizing entries in PepBank by adding an interactive retrieval heat map that allows to interactively drill down to the relevant entries. The user can add relevance constraints with respect to these common categories and immediately observe their effect on the result set. We sought to address the problem of determining the relevance to each category by integrating collection of expert knowledge and automated classification into the retrieval workflow.

No single annotator can be an expert in all disciplines and approaches published. Moreover, disagreement among experts and nomenclature specific to different scientific fields make it non-trivial to create annotation guidelines. Community voting is one paradigm well suited to overcome this problem. The central idea of our approach is to leverage annotations contributed by the users and utilize it as feedback to improve automatic classification. In our approach, annotation (also called labelling) is extremely simple for the user and amounts to voting (yes/no) on whether the current classification is correct when examining an individual entry.

PepBank currently contains nearly 20,000 peptide sequence entries and, like most other biological databases, is constantly growing. Most of its contents are automatically extracted from the literature and lack suitable annotation. Even though MeSH terms (medical subject headings) are available for many abstracts from which the sequences are extracted, they are often not yet available for recent entries. Also, they often do not reliably capture the kind of information our users are interested in (e.g., the availability of binding data). Even when using community voting, manually labelling all the database contents is human labor-intensive and naturally would lag behind as new entries are acquired. Consequently, the amount of contributed data is usually small compared to the amounts of automatically gathered data. Machine learning represents an alternative, automated strategy, as it makes it possible to train classification algorithms on abstracts that have previously been labelled by community voting. This approach maximizes the use of community-contributed information by labelling actual entries and at the same time helping to build better models for automatic classification of unlabelled entries.

We envisioned a system that is interactive and incorporates user input. Hence, it is desirable for classification to benefit from newly labelled entries as fast as possible while making reasonable use of the available computing resources. To this end, we implemented a system that immediately responds to new labels and builds improved models for automatic classification on the fly while the system is in use.

The system uses a novel combination of heat maps and text classification methods in a Web 2.0 setting. Each of these aspects have been well studied:

Heat maps are a well-known tool in the life science community, e.g., for the visualization of microarray data [9] and search results [10]. They deliver a concise and quick overview of tabular numerical data. Extended by a set of controls to add constraints and impose a search order on the visualized data, heat maps can be used to intuitively navigate to the most relevant entries in large result sets.

Web 2.0 approaches are based on participation, self-improvement (i.e., systems that get better the more people use them) and trust in users [11]. Typically they deliver a rich user experience through the use of technologies such as AJAX. Approaches to glean knowledge in a form that has relatively little predefined structure from community participation have been successful in applications such as Wikipedia [12]. The quality of community-contributed data has been the subject of ongoing debate, but is generally considered to be adequate (for an example of a study of recovery from vandalism in Wikipedia see [13]). The United States Patent and Trademark Office is currently participating in testing a web-based system for open peer-aided patent review [14,15]. Social Web 2.0 environments are also emerging in a scientific context. In the case of the public sequence database GenBank, it is increasingly recognized that the growing volume of information paired with the inability to correct annotation errors by users other than the contributing authors and internal curators may lead to deterioration of database quality [16,17]. It has been argued that uncorrected errors in public databases can percolate as they may cause dependent studies to be based on false assumptions [18]. Community participation, such as Web 2.0 approaches, may help correct some of these errors. Entrez Gene includes the Gene References into Function (GeneRIFs), which are valuable annotation data contributed in part by the users [19]. Article stubs in Wikipedia have been automatically created using data from authoritative sources to assist in gleaning additional information from the life science community about biological entities such as genes [20] or RNA [21]. Using semantic technologies, the WikiProteins system [22] strives to combine factual knowledge with community annotation in a Wiki-like system to gather knowledge that is accessible to methods of data mining. The IDBD database collects biomarker information pertaining to infectious diseases by community collaboration of registered users [23]. The ORegAnno system [93] collects information on gene regulatory elements and polymorphisms in a collaborative way and cross-references against public repositories. Its annotation queue contains papers entered by experts or identified by text-mining methods. Other approaches extend established information retrieval systems by facilities that add a community context: The CBioC project, for example, enables community annotation of molecular interaction data [24] by providing a browser plugin that opens a window when the user visits PubMed. Automated text extraction is used by CBioC for bootstrapping the database with initial data, while allowing community users to refine annotation of contents by contributing factual knowledge or voting on classification accuracy. A recent plugin for the Cytoscape software enables user annotation of molecular interaction data from the MiMI database directly within the application [25]. Examples of other community-centered approaches include platforms related to publication of original research (e.g., PLoS ONE [26]), management of references (e.g., CiteULike [27]) and dissemination of research efforts using media like video and podcasts (SciVee [28]).

In contrast to approaches that allow collection of knowledge in unstructured form (such as Wiki-like systems) or structured representations (such as annotation of interactions), our system gathers binary votes on the relevance of entries with respect to certain categories of interest. Rating of relevance or quality of entries (such as scientific contributions, content and comments contributed by users, products, or software packages) has been adopted by many websites (e.g., plosone.org, digg.com, amazon.com, and cpan.org).

Text classification is a well-studied task in the field of biomedical literature. A large number of different learning techniques such as Naïve Bayes [29,30], rule learners [31], Bayesian networks [32], and support vector machines [33,34] has been used to classify biomedical texts, for example MEDLINE abstracts and medical reports. The state-of-the-art of text classification in the biomedical domain has been evaluated via common challenge evaluations such as the KDD Cup [35,36] and the Text Retrieval Conferences (TREC). In 2002 the KDD Cup included a task centered around finding articles that contained gene expression products warranting annotation for inclusion into the FlyBase database [37]. In 2004 the Genomics track of the TREC competition [38] included text categorization tasks that dealt with automatic assignment of Gene Ontology terms to full text biomedical documents [39]. A subtask in the BioCreative II competition in 2006 dealt with detection of articles relevant to annotation and extraction of interaction information [40]. To aid human curators, the utility of text classification for pre-annotation filtering or ranking of list of search results according to their relevance to a database annotation task has been investigated on data focusing on immune epitopes [29], protein-protein interactions [41], genetic variants of human proteins [42], and allergen cross-reactivity [43]. In text mining applications, text classification is often only the first step in finding sets of documents that are pertinent to a certain topic. It is often followed by identification of relevant entities in the text (named entity recognition; NER) and automatically finding semantic relationships between these entities (information extraction; IE). An overview of text mining approaches and applications is given in [44] and [45]. In general, performance of text classification is domain-specific and depends on factors such as the training data, pre-processing, and the selection of a supervised learning algorithm. Even choosing a sensible measure of performance depends on the requirements at hand, e.g., whether false positive or rather false negative rates are to be minimized. Hence, it is important to thoroughly test different established approaches for each new application. A recent survey of opinions about the future prospects and challenges in biomedical text mining among leading experts in the field [46] identified the need for intuitive and easy-to-use interfaces for the biological end user and an increasing interest in community-based annotation among the major themes.

The goal of this study was to develop methods to speed up navigation in large automatically extracted biomedical datasets by offering users a way to prioritize search results according to information that previously had to be manually found in the associated abstracts. Rather than ensuring correctness of each contributed or predicted annotation, we aimed to make the confidence of the prediction transparent to the user, so that they are in the position to decide which entries in the result set they should manually inspect first.

## Results and Discussion

Information retrieval from textual databases using thesaurus terms or full-text search has a number of limitations: Often thesaurus terms have not yet been assigned to recent entries. Another issue arises for highly interdisciplinary subjects that span broad fields (e.g., molecular imaging) and cannot be adequately described by one single term. Full-text search in abstracts complements searching with thesaurus terms in these cases. However, full-text search always requires the user to know in advance what search terms are relevant. Hence, entries containing relevant terms that the user is not aware of can be missed. For example, it is not easy to come up with a comprehensive list of all search terms that signal the availability of binding data in an abstract. Hence, although useful, thesaurus and full-text search in abstracts alone do not fully cater to the needs of efficiently finding relevant entries in search results that have not been annotated by experts, which calls for a novel approach.

### Navigating through search results using heat maps

**Figure 1:**
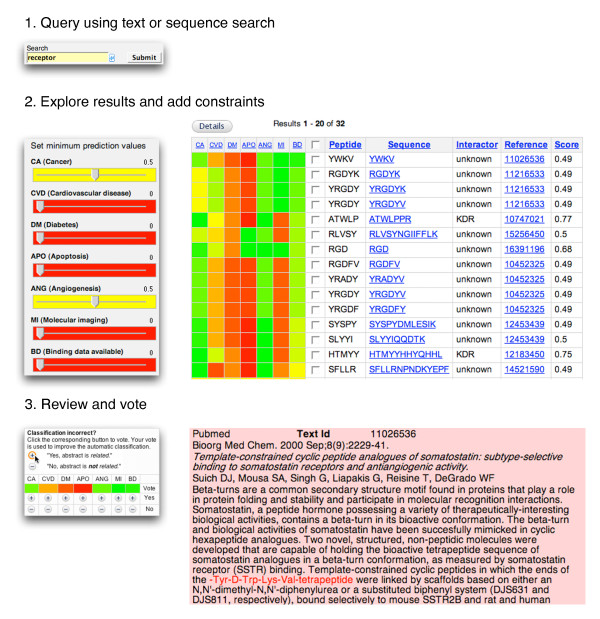
**Screenshot of the retrieval heat map**. As an example to illustrate the utility of our system, we show how the user can search for peptide sequences that bind to receptors with known affinities, and are related to cancer and angiogenesis. The user searches for "receptor" and finds 4222 results, which is far too many to sort through manually. The heat map in the results view is displayed together with additional columns (sequence, interactor, reference, and score). These columns are either automatically populated or manually curated [1]. For example, an *unknown *entry in the interactor column means that the biological interactor of the peptide has not yet been determined by manual curation of the associated abstract. The heat map shows how each entry is annotated (by vote ratios) or automatically predicted (if no votes exist) to relate to each category. To prioritize the results, the user selects the entries related to cancer and angiogenesis by setting the threshold for the "cancer related" category to 0.5, which reduces the result set to 477 entries, and for the "angiogenesis related" category to 0.5, which reduces the result set further to 32 entries. By clicking on the BD column header, entries are sorted by the availability of binding data. Clicking on the top hit (YWKV) leads to an abstract with the peptide sequence that satisfies the user constraints and the initial query. The sequence corresponds to one of the analogs of somatostatin, many of which are used in cancer treatment and diagnosis, as well as inhibitors of angiogenesis. In the entry view a voting box is offered for the user to cast a vote to either confirm or reject the offered classifications.

Our approach to improve navigation in search results is to present the user a heat map. In the heat map each row represents a target entry (a peptide interaction in our example) and each column represents a category of interest. Each cell then represents a prediction value (class probability estimate) for a peptide entry to relate to a category, converted into a color between red (*unrelated*) and green (*related*). The interface and an example user workflow are shown in Figure 1.

Controls on the web page allow exploration of the result set by sorting on individual categories, e.g., bringing all entries to the top that are related to cancer. Sorting on multiple categories (e.g., ordering by availability of binding data for entries having the same probability estimate for being cancer-related) can be accomplished by first sorting on the category for the secondary order (binding) before sorting on the primary category (cancer). To allow for an even more finely grained control of the search results, we added sliders to the interface that can be used to choose a minimum threshold for each category, e.g., to only display entries that contain binding data. This allows adding constraints after the query has been made. The interface is also highly responsive: Immediate feedback enables the user to directly understand the effect that adding a constraint has on the result set, which is not possible at query time.

### Exploiting community knowledge by voting

The values shown in the heat map are generated by either of two mechanisms: First, we allow external users of the database (and not only the internal database curators) to cast votes on each entry. As disagreement among users is possible, the value for each heat map cell is calculated as the ratio of "related" votes to the total number of votes for the entry (related and not related to the category). The values shown in the heat map are these ratios (if votes exist). Second, we use the class probability estimates of text classification if votes do not exist, as is currently true for the majority of the entries.

The rationale behind letting users vote without restrictive access control is to lower the hurdle of contributing knowledge as much as possible. Although it can be argued that this might lead to a diminished quality of labelling, we believe that the benefits outweigh potential drawbacks. Experts from different domains can contribute knowledge in a very direct fashion. Even if there is disagreement among voters, the data are not completely invalidated since disagreement is made visible by a change in color. The majority vote determines whether an entry is on the green or red side of the spectrum. Second, even weakly labelled data speeds up the retrieval process: Community gathered votes are strictly separated from data in PepBank. Having the right label means that a relevant entry might be found more quickly by using the heat map. At the same time, no data that are labelled incorrectly in the heat map are lost as they are still accessible via conventional search options like full-text search in abstracts, advanced search on single database fields, and BLAST [47,48], or Smith-Waterman [49,50] sequence similarity searches. The user can also make a trade-off between precision and recall by choosing a cut-off for further inspection of results. The interactive slider mechanism enables the user to instantly observe the effect of the chosen cut-off value on the result set.

Each vote is recorded separately with a time-stamp and client information. This allows for removal of individual votes in the event of vandalism, which is an extreme case of incorrect labelling. However, most machine learning schemes (see below) do not assume that the class labels (votes) are always correct. Rather, they are based on the assumption that classes are noisy or assigned according to a conditional probability distribution (see, e.g., [51] and [52]).

### Application of established learning techniques

**Figure 2:**
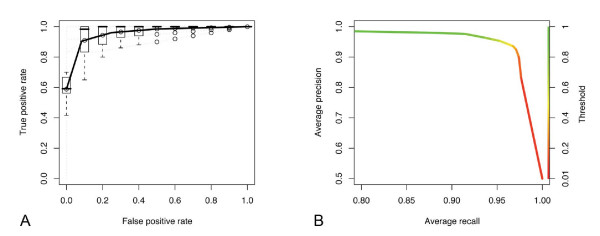
**(A) ROC plot for the angiogenesis category**. Dotted lines represent 10 individual ROC curves, which are summarized by averaging vertically, and shown as a solid line with box plots. **(B) Precision-recall plot for the angiogenesis category**. Color shows prediction value thresholds. The color key (right y-axis) has been calibrated to the actual colors used by the heat map in the GUI. For example, the prediction value threshold of 0.5 (yellow region) corresponds to a recall of 0.96 and a precision of 0.94.

To evaluate the performance of different classifiers on our domain of application we created training sets for each of the seven categories (see below). While good performance is critical for the choice of a classification algorithm, it was not the only criterion for our application. We sought an algorithm that also yields class probability estimates that could be used as confidence guides for the user and is fast enough to be used in the database-driven setting to continually rebuild classification models upon addition of user-contributed votes. We benchmarked a number of established techniques on the seven training sets using 10 iterations of 10-fold cross validation to find algorithms that perform well on all categories present in the current system. The input texts were transformed into a bag-of-words representation and stop words were removed. After Lovins stemming [53], features were transformed into TFIDF values before being presented to the learners. We used the F-Measure for related entries (e.g., related to cancer) as the performance criterion, with corrected resampled paired t-test [54] to assess statistical significance (see methods). We found (Tables 2, 3 and 4) that no learning scheme performs consistently better than bagging of the J48 variant of C4.5 decision tree learners (see methods), which achieved a performance of 91-98% (F-measure) on the benchmarking data. Figure 2A shows the ROC analysis [55] for the angiogenesis category. Bagged J48 is reasonably fast: updating the heat map for the entire PepBank through the daemon (see sections below) takes at most five minutes per category on our production system (see methods). It also offers the advantage that bagged prediction values have a sufficiently broad distribution suitable for graphically presenting the results in a heat map (Figure 1), rather than a simpler yes/no prediction. Figure 2B shows the utility of these prediction values for the angiogenesis category by combining a precision-recall plot with the actual color values used in the heat map for displaying search results. Note that the most notable change in the trade-off between precision and recall occurs in the yellow region (prediction value ≈ 0.5) where it would be naturally expected. Thus, the class probability estimates produced by this classification setup serve as useful guidance for the end user to assess prediction confidence.

**Table 2:** Performance of established classification techniques compared to bagged J48.

	**Bagged** **J48**	**Naïve** **Bayes**	**SVM**			**k-Nearest Neighbor**		**Trees**		**PART**
**Dataset**			**Polynomial kernel degree**					**J48**	**Random** **Forest**	
			**1**	**2**	**3**	**1**	**3**			
CA	**92.1 (5.0)**	87.1 (8.0)	91.4 (5.7)	89.4 (6.6)	79.4 (5.9) •	66.9 (10.3) •	73.2 (9.2) •	88.7 (6.5)	81.7 (8.0) •	86.3 (8.5)
CVD	**91.8 (4.6)**	89.5 (7.2)	92.5 (4.7)	89.2 (5.1)	83.6 (6.5) •	56.9 (12.4) •	64.3 (14.3) •	90.5 (5.7)	83.9 (6.7) •	85.6 (6.8)
DM	**96.4 (3.6)**	83.3 (8.3) •	95.2 (3.8)	89.0 (5.2) •	78.4 (4.0) •	74.4 (6.9) •	75.9 (6.3) •	96.5 (4.5)	83.1 (6.8) •	96.2 (4.7)
APO	**98.3 (3.1)**	94.8 (4.8)	98.3 (2.6)	96.3 (4.0)	41.7 (22.1) •	24.2 (18.4) •	33.4 (18.6) •	98.1 (3.8)	86.9 (7.4) •	97.2 (3.7)
ANG	**95.3 (4.2)**	94.3 (4.5)	95.3 (4.1)	93.4 (4.4)	91.5 (7.0)	47.7 (18.3) •	48.5 (18.5) •	94.0 (5.4)	89.5 (5.7)	91.3 (6.1)
MI	**94.5 (3.9)**	91.0 (5.6)	94.5 (4.1)	89.9 (4.4)	77.1 (4.0) •	73.4 (4.9) •	71.9 (4.1) •	92.1 (5.0)	85.4 (7.1) •	92.2 (4.8)
BD	**90.7 (5.3)**	87.1 (6.2)	91.1 (5.4)	88.0 (5.6)	75.8 (4.5) •	69.7 (2.9) •	67.4 (1.3) •	86.7 (6.4)	82.3 (7.8)	83.4 (7.4) •

F-Measure (in %) and standard deviation in parentheses. • statistically significant degradation. No statistically significant improvement was observed.

**Table 3:** Performance of meta classification techniques compared to bagged J48.

	**Bagging**		**AdaBoost**		**MultiBoost**	
**Dataset**	**J48**	**PART**	**J48**	**PART**	**J48**	**PART**
CA	**92.1 (5.0)**	91.9 (5.5)	93.1 (5.4)	92.0 (5.9)	92.5 (4.8)	91.3 (6.0)
CVD	**91.8 (4.6)**	91.1 (5.6)	91.9 (5.0)	90.5 (5.8)	91.9 (5.0)	91.6 (5.4)
DM	**96.4 (3.6)**	96.2 (3.7)	97.3 (3.6)	97.0 (3.6)	97.0 (3.3)	96.9 (3.5)
APO	**98.3 (3.1)**	98.3 (3.1)	98.9 (2.5)	98.3 (2.8)	98.6 (2.7)	97.9 (3.4)
ANG	**95.3 (4.2)**	95.6 (4.2)	96.0 (3.6)	94.9 (4.1)	95.1 (4.2)	95.1 (4.3)
MI	**94.5 (3.9)**	94.6 (3.5)	95.4 (3.8)	95.1 (3.9)	94.9 (4.1)	94.4 (3.8)
BD	**90.7 (5.3)**	89.6 (6.3)	91.4 (4.3)	90.1 (6.1)	91.2 (5.1)	89.6 (6.1)

F-Measure (in %) and standard deviation. No statistically significant degradation or improvement was observed.

**Table 4:** Performance of bagged J48 on all categories.

**Dataset**	**F-Measure**	**Precision**	**Recall**	**Accuracy**
CA	92.1 (5.0)	86.8 (8.1)	98.7 (3.5)	91.3 (5.9)
CVD	91.8 (4.6)	87.2 (7.4)	97.5 (5.1)	91.2 (5.2)
DM	96.4 (3.6)	94.4 (6.1)	98.7 (3.1)	96.2 (3.9)
APO	98.3 (3.1)	96.9 (5.7)	100 (0.0)	98.2 (3.5)
ANG	95.3 (4.2)	94.2 (6.1)	96.8 (4.7)	95.2 (4.3)
MI	94.5 (3.9)	91.3 (6.5)	98.3 (3.6)	94.2 (4.3)
BD	90.7 (5.3)	86.8 (7.5)	95.6 (6.5)	90.1 (5.8)

Some classifiers such as support vector machines (SVM) had F-measures comparable to those of the bagged J48 and were faster, however, the classifier output could not be consistently translated into meaningful class probability estimates spreading over the whole range between zero and one. One way to achieve this would be to convert the distance of each classified instance from the optimally separating hyperplane into a prediction value [56]. Other classifiers, such as k-nearest neighbor, performed consistently worse than bagged J48, perhaps reflecting the relative sparseness of the training sets.

### The utility of user votes as training data for automatic classification

Allowing users to vote without access restrictions eases collection of labelled training data at the cost of erroneous and inconsistent labels. Some inconsistency in user-contributed information is due to diverging expert opinions that take the form of probability distributions rather than a single standard of truth. The PepBank system shows such disagreement by converting the ratio of user votes (or bag votes of the ensemble of classifiers) into a probability estimate. Such "debated" entries are presented to the user as cells with yellow hue in the heat map.

However, some inconsistency is due to incorrect or negligent voting behavior. To estimate the impact of votes that are incorrect with respect to a gold standard, we created an additional set of 200 labelled abstracts for the "binding" category. Each entry in the data set was independently labelled by two curators, who adhered to an annotation manual that was established before the start of annotation. Inter-rater agreement was calculated to be 98% (percent of votes with the same label assigned). Labels from both curators were then consolidated into a gold standard and disagreement on the four abstracts with different labels resolved using the annotation manual. The final data set contained 63 abstracts that were labelled as containing binding data. (i.e., 31.5% of examples were in the positive class). Note that a classifier that performs random guessing (with both classes equally likely) is expected to achieve an F-measure of 38.7% on this data set (see methods section).

Next, we simulated the addition of user-contributed votes with different error rates to a baseline set used to provide the classifier with initial training data. To obtain robust estimates of performance and variance, for each error rate, the simulation was carried out using 10 different splits of the gold standard into simulated user votes and test data. Specifically, for each of the 10 runs, three data sets were automatically created in the following way: First, 100 baseline votes were sampled from the set of 240 labelled abstracts used in the previous investigation that compared performance of different algorithms (Tables 2, 3 and 4). Resampling of the base votes was carried out for each run to ensure that subsequent performance changes upon addition of votes were not dependent on a particular choice of base votes. Second, a set of 100 abstracts was sampled from the gold standard to simulate user-contributed votes with controlled error rates. This was done to ensure that the simulation results were not dependent on a particular sample of simulated user votes. Third, the remainder of the gold standard was used as a test set for this run. Sampling from the gold standard was done on a per class level, so that the simulated votes had the same number of positive and negative examples (+/- 1) as the test set.

All sampling was done without replacement. For the simulation of user votes, we made the assumption that the gold standard indeed represented the standard of truth. To simulate erroneous votes, labels from the gold standard were flipped with a probability representing the simulated error rate before addition to the training set. For example, an error rate of 0.1 means that a label from the gold standard was converted from "contains binding data" to "contains no binding data" and vice versa with a probability of 0.1 before addition to the training set.

From the data sets (baseline, user votes, test) created for each run of the simulation individual training sets were automatically created in turn by successively adding labelled abstracts from the set of simulated user votes to the baseline set. For each training set the classifier was retrained as in the online version of PepBank and evaluation was carried out on the test set for this run. Thus, each training set consisted of the base votes (100 abstracts) and a variable additional number of simulated user-contributed votes (0-100 abstracts, with simulated errors).

**Figure 3:**
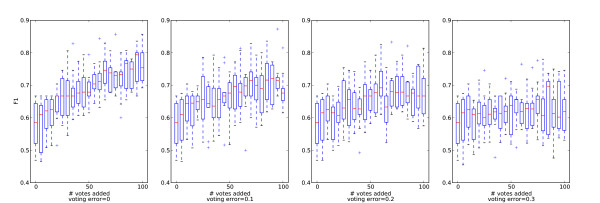
**Box plots showing the effect of adding simulated user votes**. Each subplot shows the effect of adding 100 simulated user votes with controlled error rates (0-0.3) to a training set with 100 entries for the binding category. The votes are added in increments of 5 votes, the training set for the classifier producing the first box plot has 0 votes added (only the resampled 100 base votes), the next one has 5 votes added, etc. The last box plot shows the performance after adding 100 simulated user votes to the base votes.

The box plots in Figure 3 show the development of classifier performance as simulated user votes are added (F-measure for the related class). While inconsistent votes have a negative effect on the classification performance in general, voting helps to significantly increase classification performance even if one assumes voting error rates as high as 20%.

### Adding text classification capability to an existing database: a database-driven approach

One key requirement for our classification system was to adapt to new user-contributed data dynamically and fast. Also, integration of text classification into the database was desirable since it simplifies the system and renders the user interface completely independent from the machine learning part. Inductive databases, systems that natively support data mining operations, would be an ideal solution for this application. Theoretical and practical attempts have been made to define formal requirements for inductive databases and to extend database systems by machine learning operations [57-61]. Oracle 11 g offers database-integrated text classification using support vector machines and decision trees [62]. We created a system that is similar in spirit in that it is controlled by the database but leaves classification to a background process (daemon) running alongside of the database server.

**Figure 4:**
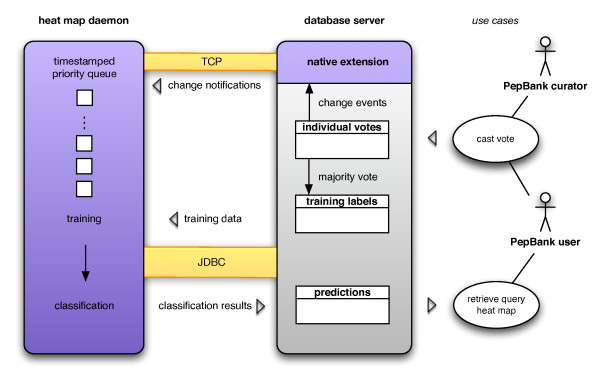
**System architecture for community voting and database-driven classification**. A PepBank user enters a vote through the web interface or a PepBank curator uploads votes in batch. Individual votes are aggregated and training labels assigned according to the majority vote. A native database extension sends a notification to the classification daemon. A task is placed in the queue, and the category is scheduled for reclassification. Upon completion, the classification results are read into the database and stored as the new prediction values. They are seamlessly displayed to the user through the web interface. The entire process is independent of the number of votes cast per category and takes less than 5 minutes per category.

The classification system is controlled by the database through a native extension. Update events can thus be issued from within the database and triggers can be built that automatically notify the daemon that changes have occurred. The system architecture is outlined in Figure 4.

Based on the benchmarking results, we selected bagged J48 decision trees to classify unlabelled entries in PepBank. The daemon trains the classifier on the initial expert-curated training sets (used for Tables 2, 3 and 4) and on those database entries that have received votes from the users. The trained classifier is then used to classify all entries that have not yet received votes. As more votes are being cast, the model for classification is expected to improve.

Peptide entries (whether curated or automatically extracted from the literature) are classified on the basis of their associated abstracts using the same pre-processing steps of stop-word removal, Lovins stemming, and TF-IDF transformation that were used during benchmarking (see methods section). For example, the text fragment "Tumor-induced angiogenesis can be targeted by RGD (Arg-Gly-Asp) peptides, [...]" (PMID 16391196) would yield the tokens {*angiogenes*, *arg-gly-asp*, *pept*, *rgd*, *targes*, *tumor*} after stop-word removal and Lovins stemming. TF-IDF values are then calculated on the basis of the token frequencies. The bag-of-words representation of an abstract is a sparse vector containing the TF-IDF values of the observed tokens. Note that the bag-of-words representation of an abstract does not change when new votes are cast. Voting only affects the class labels of abstracts presented to the classifier during training.

Each user vote and associated meta-information are recorded separately in a database table. This enables deletion of individual votes from a certain origin (e.g., a specific client) or adding a manually uploaded batch of training data independently from other votes.

The class probability estimates in the heat map are updated through the following mechanism: First, whenever a new vote is cast, triggers in the database aggregate individual votes into a single entry for each abstract and each category, containing the vote counts for both classes. This enables the classification daemon to read the counts for each entry with a single inexpensive query. Second, the triggers also call a database extension that notifies the classification daemon that a change in the training data for a specific category has occurred. Finally, the daemon schedules the category for reclassification by putting a time-stamped entry into a priority queue. If another request for reclassification with an earlier time-stamp exists that is not yet being processed, the new request is ignored and the time-stamp of the earlier entry is updated to ensure non-redundant classification.

Whenever there are reclassification requests in the queue, the daemon asynchronously retrieves the next entry from the priority queue and reads the training data for the corresponding category: the set of labelled abstracts and the vote counts for each class. If there is disagreement among the user votes for an entry, the majority vote is used to determine the right class assignment for the training data. If the same amount of votes has been cast for both classes, the entry is excluded from the training data. After the classifier has been trained, the daemon updates the class probabilities for entries that have not received any votes yet.

The setup described above makes efficient use of resources since no redundant classification is done. At the same time the heat map benefits from new votes as fast as possible. The scheduler ensures that the waiting time from casting a vote to the heat map update is bounded by *O*(#*categories • t*_*c*_) where *t*_*c *_is the maximum time for reclassification of a single category, and is not dependent on the number of concurrent votes being cast. The notification of the daemon and the use of a priority queue scale well when casting thousands of votes during batch uploading.

### Future directions

The current system has a fixed set of categories for classification, which were determined by the needs of our users. However, since the system is scalable, it could be worthwhile to allow dynamic creation of categories by the users themselves in the future.

Even with community voting the generation of training data still requires human labor making it relatively expensive to obtain compared to the millions of unlabelled abstracts in MEDLINE. The availability of large amounts of unlabelled data is a common theme in bioinformatics. Recently, semi-supervised or transductive learning algorithms have received attention in machine learning [63-65]. In semi-supervised learning, the learner benefits from unlabelled examples by capturing information about the domain-specific distribution of examples. This is motivated by the so-called cluster assumption [66], which states that nearby instances in the example space are likely to have the same label and that classification boundaries are unlikely to run through regions densely populated by examples. It has been shown that semi-supervised learning can lead to performance improvements especially when dealing with small amounts of labelled data [66]. The current application with its large amounts of unlabelled training data seems to be a good target for transductive machine learning methods and we are considering benchmarking different transductive learners on the abstracts in PepBank. Finally, we believe that the problem would be suitable for methods from online learning [67].

Currently, only abstract texts but not titles or MeSH terms are used for classification. The reason for leaving the latter out is that many newly submitted entries, which contain information relevant to PepBank, have not yet been assigned MeSH terms yet. Using title words, MeSH terms, journal titles, and author names for classification of MEDLINE abstracts can lead to performance gains [29]. We are planning to address this in a future release.

Stemming is an important part of pre-processing text for classification. Whether stemming is beneficial in terms of classification performance highly depends on the domain of application and the stemming algorithm used. Han et al. [68] and Wang et al. [29] report that the use of standard stemming algorithms might not be suitable for texts in the biomedical domain with terms vastly more complex than in everyday English. For our future work we are looking into more rigorous ways to evaluate different stemming algorithms for our application and into testing other stemming methods such as language independent frequent substrings, which have been shown to be well-suited for a variety of biomedical text classification tasks [68].

## Conclusion

Machine learning can be used to tackle the problem that, even when using community voting, data available in biomedical databases often outnumber the amount of manually contributed meta information. Furthermore, machine learning can be used in an interactive setting to take advantage of community-contributed information as fast as possible. Our approach to implement a database-driven machine learning system is independent of the presentation layer and scales well with frequent changes in the user-contributed data that are used to build automatic models for classification. In our application, bagging allows ensembles of J48/C4.5 decision tree learners to deliver meaningful predicted class probabilities that are helpful to visualize the trade-off between precision and recall in heat maps for navigation. The setup we describe can be readily applied to other databases that store textual data to enhance navigation without requiring changes to the respective underlying database models.

## Methods

### Pre-processing of text

#### Stemming

Stemming removes the inflectional affixes of words to reveal their stem. This technique has been widely used in text classification. Stemming not only reduces the feature space but also combines words in different inflectional variants into a single feature. Lovins stemming was applied to pre-process MEDLINE abstracts prior to benchmarking [53]. The same pre-processing step is also used in the production system.

#### TFIDF

Term frequency and inverse document frequency are two well-accepted measures for unsupervised feature selection and pre-processing of bag-of-word features in text classification. Although we did not perform any feature selection on our training set, TFIDF transformation was used to transform the data set prior to presenting it to different classifiers for benchmarking. Naive Bayes has been shown to benefit from the additional information in TFIDF transformed feature values [69]. The frequency f of a certain term i in a document j of the training corpus is called the term frequency *f*_*ij*_. The inverse document frequency gives a measure of interestingness of the term by taking into account how many documents d in the training set D contain it:

. These two measures were combined to transform the word frequencies in the bag-of-words data set: *TFIDF*(*term*_*i*_, *document*_*j*_) = log(1 + *f*_*ij*_)·*IDF*(*term*_*i*_) [70].

### Evaluation

#### Construction of training sets

Because PepBank contains only abstracts with peptide sequences, it only includes a very small subset of MEDLINE and thus may have a biased representation of some terms useful for classification. We therefore sampled the training set from a broader distribution of MEDLINE entries beyond PepBank. We first used fairly broad queries for each category to enrich the sets for related or unrelated abstracts correspondingly, and then performed manual labelling of each abstract. For each category, we manually labelled 120 MEDLINE abstracts for each of the "related" and the "unrelated" classes by a single annotator. Note that the categories are not mutually exclusive, that is an entry can be related to several categories such as cancer and angiogenesis at once. However, as different classifier instances are produced for each category, this does not present a problem.

For the simulation of user voting we created a different set of 200 abstracts for the binding category that was annotated by two curators. To serve as guidance during the annotation process, an annotation manual was established before the start of annotation.

To ensure reproducibility of results, the datasets' PMIDs, labels, and the annotation manual are available from the authors on request. A simple voting guide for Pepbank users (rather than the more exhaustive annotation manual for curators) is available on our web site .

#### Cross-validation

In n-fold cross-validation the data set is split into *n *disjoint sets or folds. The classifier is then trained on *n *- 1 folds and tested on the remaining fold. This process is iterated so that testing is carried out on each fold exactly once. Thus, the classifier can be evaluated on the entire data set without exposing class information of test instances during training. We carried out the benchmarking experiments using 10-fold cross-validation.

#### Precision, recall, and F-measure

Precision and recall are two metrics that are often used to evaluate the performance of information extraction systems. Precision is the probability that a positively classified example is indeed a true positive: , whereas recall describes the probability that a positive example in the test set is indeed classified as positive: . Here, TP and FP are the numbers of true and false positives and TN and FN the numbers of true and false negatives, respectively. An abstract was considered a true positive, if it was related to a certain category and correctly classified by the learning algorithm. A true negative example is unrelated to the category in question and correctly predicted as unrelated by the classifier. We used the F1-measure, which is the harmonic mean of precision and recall with both equally weighted:  to give an overall figure of merit [71]. Let  be the fraction of positive examples in a test set. A classifier that merely performs random guessing will yield an expected precision of *E *[*prec*_*guess*_] = *p*_*positive *_on this set, since the examples it labels as positives will be a random subset and thus be true positives with a probability of *p*_*positive *_. The same classifier would (assuming both classes to be equally likely) achieve an expected recall of *E *[*rec*_*guess*_] = 0.5, since by chance it is expected to identify half of the positive examples correctly. Combined, this yields an expected F1 measure of  for random guessing.

#### Benchmarking

Benchmarking was performed using Weka Experimenter [72] on a 64 node HP-XC cluster system with a MySQL [73] server instance to collect the results. Statistical testing was carried out using the corrected resampled paired t-test [54] with a significance level for comparisons of 0.05. Bonferroni adjustment for the number of benchmarked learning schemes was applied [74]. ROC and precision/recall plots were produced using thresholded classification data from 10 cross validation folds using R [75] and the RWEKA [76] and ROCR [77] packages.

#### ROC plots

ROC (Receiver Operating Characteristics) graphs have been used in signal detection, medical diagnostics, and machine learning. These two-dimensional plots are used to visualize the trade-off in classifier performance between true positive rates (y-axis) and false positive rates (x-axis) [55]. Evaluation of a classifier on the test set produces an (FP rate, TP rate) pair that can be represented by a single point in the plot. The point in the upper left hand corner of the plot (0,1) corresponds to a perfect classification. If class probability estimates exist (as in our application), points for ROC curves can be produced by choosing different threshold values. For each threshold value the class probability estimate is considered: If it is larger than the threshold, the positive class is predicted. Otherwise, a negative class label is assumed. TP and FP rates are then calculated according to this choice of the threshold. A random classifier that performs only guessing is expected to show a ROC curve that corresponds on average to the diagonal running from the lower left (0,0) to the upper right hand corner (1,1) of the plot. ROC plots are well suited for scenarios of unbalanced classes and show how a classifier performs when tuned for either conservative (high precision) or liberal (high recall) prediction.

### Classification

#### J48/C4.5

J48 is an implementation of Quinlan's C4.5 release 8 decision tree learner [78] provided by Weka. Decision trees essentially define a series of tests on the attributes of each instance that is classified. Each internal node describes a test on a specific attribute. The children represent possible values or a range of values of the test attribute. A child node can either be the root of a new subtree that describes further tests or a leaf node that represents a class label. C4.5 performs induction of a decision tree given a set of training examples. Decision trees are built top-down in a divide-and-conquer fashion. The algorithm selects the best attribute for the next test by evaluating the reduction in entropy with respect to the distribution of class labels that splitting the training set on each attribute would have. After the decision tree has been built, C4.5 applies pruning to reduce complexity of the tree and avoid overfitting. J48 was run using standard parameters with enabled subtree raising, a confidence factor of 0.25 for pruning and the minimum number of instances per leaf set to two.

#### Bagging

Bootstrap aggregating (Bagging) [79] is an ensemble technique to create multiple versions of a classifier and use them to build an aggregated classifier. The different versions are created by bootstrap sampling of the training data. In a regression setting bagging averages over the numerical prediction values made by members of the ensemble. In classification the majority vote is used. However, the ratio of the votes cast by different members of the ensemble can be used as a measure of confidence, which loosely mimics agreement among human experts. In our implementation the class probabilities from different versions of the classifier were aggregated to obtain the bag vote and the aggregated class probability estimate. Bagging improves classification performance if small changes in the training data among different bootstrap samples cause large perturbations in the constructed classifier models, as is the case in decision tree learners. In fact, Quinlan shows that bagging C4.5 classifiers leads to significant performance improvements over a number of diverse data sets [80].

#### Other supervised learning methods

Naive Bayes [81], k-nearest neighbor [82], and support vector machines [83-85] are well-established classification methods. PART [86] is a rule learner that repeatedly generates rules from partial C4.5 decision trees and does not employ any post-pruning. Boosting converts weak PAC ("probably approximately correct") learners [87] into strong PAC learners. We used the Adaboost.M1 algorithm [88]. Here, a sequence of weak learners is applied to sequentially modified weighted samples of the training data. Examples in the training data that are misclassified in the preceding iteration gain higher weights in the next iteration. Likewise, correctly classified examples have their weights lowered. Thus, each new classifier is expected to improve performance on those examples that were misclassified by previous classifier instances in the chain. Decision trees have been argued to be well suited baseline learners for boosting [89]. It is also noted that although performance of boosting is higher on average compared to bagging, it is also more variable and prone to degradation on some data sets [80]. MultiBoosting [90] is an extension to AdaBoost that combines it with wagging, a variant of bagging [91].

#### Implementation details

The classification daemon was implemented in Java using the Apache Commons Daemon [92] classes, which were originally part of the Jakarta Tomcat application server. This provides the basic infrastructure for running as a detached server with proper handling of standard Unix signals. The daemon implementation makes use of the Weka classes for machine learning. MySQL server [73] was extended by a user-defined function (UDF) to enable notification of the classification daemon via TCP. The production system consisting of PepBank and the classification daemon runs on a Fedora Core 8 Linux virtual machine running on an HP DL320 host with two 3 GHz Xeon processors, allocated 1 GB of RAM.

## Authors' contributions

TD conceived, implemented, and benchmarked the classification system and wrote the body of the manuscript. DG implemented the GUI. TD, MP, DG and TS designed the GUI and performed user testing and QA. SK contributed to the machine learning setup, benchmarking, and revised the manuscript. RW conceived the project, proposed visualization using heat maps, financially supported investigators, and co-wrote the manuscript. TS created the training sets, co-wrote the manuscript, and provided overall guidance of the project. All authors read and approved the manuscript.
